# Spermicidal Activity of the Safe Natural Antimicrobial Peptide Subtilosin

**DOI:** 10.1155/2008/540758

**Published:** 2008-10-09

**Authors:** Katia E. Sutyak, Robert A. Anderson, Sara E. Dover, Kenneth A. Feathergill, Alla A. Aroutcheva, Sebastian Faro, Michael L. Chikindas

**Affiliations:** ^1^Department of Microbiology and Molecular Genetics and Department of Food Science, Rutgers, The State University of New Jersey, New Brunswick, NJ 08854-8097, USA; ^2^Department of Obstetrics and Gynecology, Rush University Medical Center, Chicago, IL 60612, USA; ^3^The Royal Institute of Technology, 100 44 Stockholm, Sweden; ^4^Health Promoting Naturals, Inc., Highland Park, NJ 08904, USA; ^5^The Women's Hospital of Texas, Houston, TX 77054, USA

## Abstract

Bacterial vaginosis (BV), a condition affecting millions of women each year, is primarily caused by the gram-variable organism *Gardnerella vaginalis*. A number of organisms associated with BV cases have been reported to develop multidrug resistance, leading to the need for alternative therapies. Previously, we reported the antimicrobial peptide subtilosin has proven antimicrobial activity against *G. vaginalis*, but not against the tested healthy vaginal microbiota of lactobacilli. After conducting tissue sensitivity assays using an ectocervical tissue model, we determined that human cells remained viable after prolonged exposures to partially-purified subtilosin, indicating the compound is safe for human use. Subtilosin was shown to eliminate the motility and forward progression of human spermatozoa in a dose-dependent manner, and can therefore be considered a general spermicidal agent. These results suggest subtilosin would be a valuable component in topical personal care products aimed at contraception and BV prophylaxis and treatment.

## 1. INTRODUCTION

Subtilosin is a ribosomally-synthesized cyclopeptide produced by *Bacillus subtilis* ATCC 6633 and a recently identified natural isolate of *Bacillus amyloliquefaciens* [1, 2]. This protein has a unique structure among bacteriocins [3], and possesses antimicrobial activity against a variety of pathogenic organisms, including *Gardnerella vaginalis*, *Listeria monocytogenes*, and *Streptococcus agalactiae* (Group B *Streptococcus*) [2]. The ability to inhibit the growth of *G. vaginalis* is of particular importance as it is one of the primary causative agents for bacterial vaginosis (BV), a common condition found in up to 30% of women in North America [4]. BV is characterized by the replacement of normal vaginal lactobacilli with anaerobic bacteria (e.g., *Prevotella*, *Bacteroides*, and *Mobiluncus*) and mycoplasmas, as well as a several log increase in overall bacterial growth [5–7]. BV is asymptomatic in approximately one half of all cases, but is associated with a wide variety of symptoms and problems ranging from complications with pregnancies (i.e., preterm births, low fetal birth weight) to the development of pelvic inflammatory disorder [7–11]. Recent studies have estimated that nearly one in three women in the United States (29.2%) suffer from BV, with varying prevalence according to ethnicity and education levels [4]. One of the most troubling aspects of BV infection is the association with an increased risk of several sexually transmitted diseases (STDs), including chlamydia, herpes, gonorrhea, trichomoniasis, and HIV/AIDS [12–16]. The treatments recommended by the Centers for Disease Control (CDC) are clindamycin and metronidazole administered either orally or intravaginally [17]. Following these guidelines successfully treats 60% of BV cases, but 20% of these cases return with highly developed antibiotic resistances [18–20]. In such cases, it would be extremely desirable to have an alternative form of treatment that could fully eradicate the infection.

Bacteriocins are typically divided into two major, yet diverse, classes: class I, or lantibiotics, contain unusual amino acids and are subject to extensive posttranslational modifications; and class II, the heat stable nonlantibiotics [21]. Subtilosin has a unique posttranslational structure that is unmatched among bacteriocins, which has led to speculation that it may belong in a distinct and separate class [2, 3]. Bacteriocins have been widely considered for use in medicinal and pharmaceutical applications, particularly for their bactericidal activity against multidrug resistant organisms [22]. They are especially appealing alternatives since their cost of production is so comparatively low to conventional pharmaceutical treatments. For example, commercial grade nisin costs approximately $100/lb through chemical distributors. Much attention has recently been focused on the spermicidal activity of bacteriocins due to their targeted antibacterial activity and lack of effect on human tissues. Nisin A, a well-studied class I bacteriocin produced by *Lactococcus lactis* subsp. *lactis* [23], has human spermicidal activity [24, 25], and subtilosin has spermicidal activity against boar, bovine, horse, and rat spermatozoa [26]. The results of these previous studies suggest that subtilosin may also have spermicidal activity against human spermatozoa, prompting our investigation.

Since subtilosin has proven antimicrobial activity against the pathogen largely responsible for BV, its toxicity to human tissues was assayed to determine its potential as a safe alternative remedy. For the human in vitro study, the EpiVaginal ectocervical tissue model (MatTek Corporation, Ashland, Mass, USA) was employed to examine the relationship between prolonged exposure to subtilosin and cell viability. Spermicidal evaluations were conducted to investigate the ability of the peptide to restrict or eliminate sperm mobility, leading to its classification as a spermicidal agent.

## 2. MATERIALS AND METHODS

### 2.1. Production of subtilosin

Subtilosin was prepared as previously described [2]. To prepare a cell-free supernatant (CFS), cells were removed by centrifugation (Hermle Z400K; LabNet, Woodbridge, NJ, USA) for 25 minutes at 4500 g and 4°C. The supernatant was filter sterilized using 0.45 *μ*m filters (Fisher, Pittsburgh, Pa, USA). The protein of interest was precipitated from the supernatant by adding 30% ammonium sulfate (w/v) while stirring overnight at 4°C and was resuspended in 20 mL of double distilled H_2_O. The column chromatography method described by Sutyak et al. [2] was used to purify subtilosin from the CFS, producing a near-pure isolate in the 90% methanol eluate. The antimicrobial activity of all samples was confirmed by the well-diffusion assay according to the protocol of Cintas et al. [27] with additional modifications [2]. The active fraction was concentrated to dryness using a Savant SC110 Speed Vac and UVS400 Universal Vacuum System (Savant Instruments, Farmingdale, NY, USA), then resuspended in 1.5 mL ddH_2_O.

### 2.2. Determination of protein concentration

#### 2.2.1. Column-purified sample concentration

The concentration of subtilosin in the column-purified fraction was determined using the Micro BCA Protein Assay Kit according to the manufacturer's protocol (Pierce, Rockford, Ill, USA). In brief, the assay measures the reduction of Cu^2+^ to Cu^1+^ by colorimetric detection of Cu^1+^ by bicinchoninic acid. Bovine serum albumin (BSA) was used to develop a standard curve with concentrations ranging from 0.5 to 20 *μ*g/mL; the concentration of subtilosin was calculated using the *R* value from the trendline of the standard curve graph.

#### 2.2.2. Assay of subtilosin concentration in CFS

The concentration of subtilosin in the CFS was not measurable with the Micro BCA Protein Assay due to the high level of background proteins in the solvent (MRS medium). As an alternative, the protein concentration was calculated by comparing the antimicrobial activity of known concentrations of column-purified protein to equal volumes of CFS. Five two-fold dilutions were made from the stock samples of both the CFS and the column-purified fraction. Well diffusion assays were performed using 50 *μ*L of each dilution against *Micrococcus luteus* ATCC 10420, which is commonly used as a reference microorganism for the determination of a bacteriocin's biological activity [28].

### 2.3. Determination of the presence of weak organic acids

As reported previously, the concentration of lactic acid in the CFS was measured to assess its potential effects on antimicrobial activity and cell viability [2]. The quantity of each form of the acid in the sample was measured using a commercially available D-lactic acid/L-lactic acid kit (Roche Boehringer, Mannheim, Germany), according to the manufacturer's instructions.

### 2.4. EpiVaginal ectocervical tissue model

The EpiVaginal (VEC-100) ectocervical tissue model (MatTek Corporation, Ashland, Mass, USA) was used and maintained as fully described by Dover et al. [29]. The tissues were exposed to 83 *μ*L of subtilosin CFS (∼136 *μ*g/mL) for 4, 24, and 48 hours. For exposure times over 24 hours, the tissues were aerated by placing them on two metal washers (MatTek Corporation, Ashland, Mass, USA) and fed with 5 mL of the assay medium. Double-distilled water (ddH_2_O) was used as a negative control, and was applied to cells after 6, 24, and 48 hours. A spermicidal product containing 4% Nonoxynol-9 (Ortho Options CONCEPTROL Vaginal Contraception Gel, Advanced Care Products, Skillman, NJ, USA) was used as a positive control based on its documented cytotoxic properties [30, 31]. A cream (Monistat-3, Ortho McNeil Pharmaceutical, Inc., Raritan, NJ, USA) containing 4% of the nontoxic, BV-active compound miconazole nitrate [30, 31], was used as a negative control.

Following the designated exposure times, the MTT (3-(4, 5-Dimethylthiazol-2-yl)-2, 5-diphenyltetrazolium bromide) assay was used to determine overall cell viability. The data were used to approximate an effective time (ET) that would reduce cell viability to 50% (ET-50).

### 2.5. MTT viability assay

The MTT assay was carried out according to the protocol outlined by Dover et al. [29]. Briefly, the viability of ectocervical cells after exposure to subtilosin was measured as a direct proportion of the breakdown of the yellow compound tetrazolium to the purple compound formazan, since only living cells can cause this reaction to occur [32]. Tissues were exposed to subtilosin and the two controls for several designated time points; at the conclusion of each, the liquid in the plate wells was combined with the liquid from the tissue inserts. This mixture was then assayed spectrophotometrically using a 96 well-plate reader (MRX revelation, Dynex Technologies, Va, USA) to determine the level of tetrazolium degradation.

The viability (%) of the treated tissue inserts was calculated according to an equation provided by the manufacturer: % viability = OD_570_ (treated tissue)/OD_570_ (negative control tissue). The exposure time that reduced tissue viability by 50% (ET-50) was calculated according to [*V* = *a* + b* log(*t*)] described by Ayehunie et al. [30], where *V* = % viability, *t* = time in minutes, and “*a*” and “*b*” are constants representing the viability data from the time points preceding and following 50% viability. On the whole, there is a direct relationship between the length of the ET-50 and the toxicity of the tested application (i.e., a shorter ET-50 corresponds to a more harmful compound).

### 2.6. Semen sample collection and analysis

The CFS gathered from a *B. amyloliquefaciens* culture was used to test the effect of subtilosin exposure on the motility of human spermatozoa. Initially, the CFS was diluted with normal saline (0.9%) so that 200 *μ*L of the final material was equivalent to 50 *μ*L, 100 *μ*L, or 200 *μ*L of undiluted CFS.

Two semen samples were collected on the day of experimentation. Each sample was collected by self-masturbation in a polypropylene specimen container (Fisher) prior to transport to the laboratory. Within 1 hour of collection, the samples were pooled. Total sperm count was calculated using bright field light microscopy (Olympus BX50; 400x) after dilution (1:50) of the semen in normal saline. The initial percentage of motile sperm was calculated prior to testing with a neubauer hemacytometer [33]. The determination of motile sperm % was made using randomly selected field views (400x) from a count of between 104–201 cells. Any visibly moving spermatozoa (directional or stationary) were counted as motile cells.

The percentage of forward progressing spermatozoa was subjectively determined based on the assumption that 70% of the sperm in a normal sample would behave in such a manner. The samples used in this experimentation fell into such a “normal” category.

### 2.7. Treatment of spermatozoa with subtilosin

A modified Sander-Cramer test was used to determine the effect of column-purified subtilosin on human spermatozoa motility [34]. This measured the effect of subtilosin after 30-second exposure times of 5 volumes (200 *μ*L) of the solution at each dilution (25% and 50% in normal saline, and 100%) with one volume (40 *μ*L) of whole semen. The motilities of cells from random high-magnification fields (400x) of the sample were determined in duplicate as described above.

### 2.8. Data analysis

The % motility data were arcsine transformed [35] prior to further examination. StatMost32 (version 4.1) statistical software (DataMost Corporation, Sandy, Utah, USA) was used to calculate all statistical parameters. The % values of motility were presented as averages and 90% confidence limits. Any differences between treatment groups were assessed by the Newman-Keuls multiple range test. Differences were deemed significant at the 0.05 level of confidence.

## 3. RESULTS

### 3.1. Determination of protein concentration

The concentration of subtilosin in the column-purified sample was estimated at 135.7 *μ*g/mL. The CFS and column-purified sample produced identical zones of inhibition at each dilution (data not shown); therefore, the concentrations of protein in both solutions were assumed to be equivalent. While it is improbable that a 100% yield would be attained from column chromatography, previous work has shown that protein concentrations can be precisely calculated based on the comparisons we conducted [36]. Due to the difficulty in measuring the CFS protein concentration via other assays, the chosen method was deemed the most accurate and reproducible.

### 3.2. Cell viability % and ET-50 values

After 48 hours of exposure to subtilosin, the EpiVaginal ectocervical tissues retained a high level of viability compared to the positive control, Nonoxynol-9, and the negative control, miconazole nitrate (Table 1). Due to the lack of toxicity of the antimicrobial, the ET-50 value for subtilosin could not be established since the total cell viability did not drop below 50% at any of the given time points. However, a projection of the ET-50 value is possible by an extrapolation of the data. Data presented in Table 1 can be fit to a curve described by Ln(*V*) = *a* + *b* *t* ^2^, where *a* = 4.605995356 and *b* = −0.00014151 (coefficient of determination, or *r* ^2^, = 0.9998), from which the ET-50 is estimated at 70 hours.

### 3.3. Quantitative observations of motile spermatozoa

**Figure 1:**
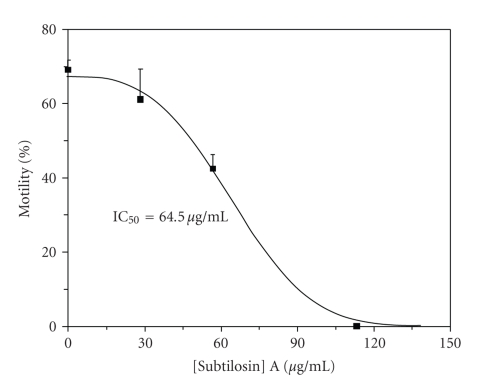
Subtilosin A immobilizes human spermatozoa in a dose-dependent manner. The percentage of motile spermatozoa in pooled whole semen was determined 30 seconds after mixing with subtilosin A, at different final concentrations, as indicated. All data were adjusted to a normal control motility of 70% and subjected to arcsine transformation before further analysis. Values are expressed as average % motility. Error bars are 90% confidence limits.

Subtilosin reduces human sperm motility in a dose-dependent manner (Figure 1). The motility of the treated spermatozoa ranged from 0 to 88% of control motility levels over the four-fold range of subtilosin concentrations. All of the subtilosin concentrations tested reduced motility compared to the control samples. The differences in the proportion of motile spermatozoa in all samples (28.3, 56.7, and 113.3 *μ*g/mL protein equivalents) were found to be significant (*P* < .05) according to the Newman-Keuls multiple range test. TableCurve 2D (ver 5.0) curve-fitting software (SPSS Scientific Software, Chicago, Ill, USA) was used to fit the data to a dose-response curve described by Ln (% Motility) = *a* + *b* [Subtilosin A]_3_, where *a* = 4.20781; *b* = −2.5814*e* − 06; and [subtilosin A] is expressed as *μ*g/mL protein equivalents. The curve had a coefficient of determination (*r*_2_) = 0.9959. The IC_50_ value, or the amount of subtilosin required to reduce the motility of spermatozoa in whole semen by 50%, was calculated to be 64.5 *μ*g/mL.

### 3.4. Semiquantitative observations of spermatozoa: forward progression

Similar to motility, forward progression of spermatozoa is reduced in a dose-dependent fashion by subtilosin. In control samples, 70% of sperm exhibited forward progression; in the presence of 50 *μ*L subtilosin this decreased to 50–70%, while 100 *μ*L caused a decline to only 10% forward progression. All forward progression was eliminated after treatment with 200 *μ*L subtilosin, with most sperm tails becoming coiled.

## 4. DISCUSSION

The *B. amyloliquefaciens*-produced bacteriocin subtilosin has proven antimicrobial activity against the vaginal pathogen *G. vaginalis*, but was not harmful to the normal and healthy *Lactobacillus* vaginal microbiota. Data from human vaginal cell viability assays convincingly demonstrated the safety of subtilosin for human applications in comparison to other accepted and available products, indicating it could be safely incorporated into personal care applications aimed at the treatment of bacterial vaginosis. Prior research in our laboratory that involved similar studies with the EpiVaginal model was also carried out in conjunction with in vivo testing of the rabbit vaginal irritation (RVI) system, which doubly confirmed the safety of another antimicrobial peptide, Lactocin 160 [29]. Therefore, we are confident that using the EpiVaginal model instead of animal testing to demonstrate the safety of subtilosin has provided reliable and valid results.

Subtilosin was also found to significantly reduce the motility of human spermatozoa in a concentration-dependent manner for all concentrations tested. The effect of subtilosin on the forward progression of spermatozoa was also observed to be a dose-dependent interaction. Serial dilutions showed a steady decline in forward progression, with all progression halted at the highest concentration tested. It was also noted that at the highest concentration, the tails of the sperm cells were curved or coiled, indicating the cells were damaged beyond a simple restriction of movement. Coiling of the cells is considered to be a sperm abnormality, and may indicate damage to the plasma membrane [37]. Tail coiling has been observed after in vitro exposure of monkey spermatozoa to methyl mercury [38]. These results suggest that subtilosin can be established as a general spermicidal agent. It is worth noting that nisin, a bacteriocin given generally recognized as safe (GRAS) status by the Food and Drug Administration (FDA), was also shown to have impressive spermicidal activity [25]. However, the allure of these results is diminished due to the fact that it also has potent antimicrobial activity against healthy vaginal microbiota (Chikindas et al., unpublished data), a strong detraction from its commercial applicability. Therefore, subtilosin's spermicidal activity, combined with its overall safety to both human tissues and healthy human microbiota, make it a highly recommendable compound for inclusion in topical BV treatments and human contraceptive products. To facilitate the process of its incorporation into contraceptive treatments, additional analysis will be done to determine the reversibility of damage done to the spermatozoa, as well as a time course assay to further elucidate the exact changes effected by subtilosin.

## Figures and Tables

**Table 1:** Ectocervical cell viability after prolonged exposure to subtilosin.

Postexposure cell viability (%)					
Exposure time (hrs)	Assay #	Subtilosin	Nonoxynol-9 (4%)	Miconazole nitrate (4%)	ddH_2_O
4	1	99.1	14.1	ND	100
	2	100	16.5	ND	100
24	1	94.8	0	58.4	100
	2	89.8	0	58.3	100
48	1	73.4	0	79.3	100
	2	70.9	0	83.5	100
